# Changes in Drug Trade

**Published:** 1877-04

**Authors:** 


### Changes in the Drug Trade.

---

MR. C. M. LYMAN having suceeeded to the interest of Mr. William Peabody, at 311 Main street, pledges himself that the reputation of the store shall be fully equal to the past in all respects in the quality of the goods, the competency of assistance, promptness and dispatch. He announces that he shall continue the name "Peabody's Drug Store," and that he has made arrangements with parties with whom Mr. Peabody has heretofore dealt in the Surgical, Chemical and Pharmecutical line as agent for Tiemann, Phelps and others, so that all the wants of the profession will be as amply provided for as they have heretofore been by Mr. Peabody.

Mr. Lyman is well known in Buffalo and vicinity as a competent and reliable druggist, and will take with him to his new location a well-earned reputation.

---

MR. A. R. DAVIDSON (late manager of Peabody's Drug Store) has purchased his up-town Drug Store, 598 Main street, corner of Chippewa, where he proposes to continue the same business, to supply the best and most reliable Drugs and Chemicals at *reasonabte prices.* Mr. Davidson is well and favorably known to the profession and by the general public, and will enjoy the confidence of all who have done business with him.

---

MR. RODENBACH has also established a new up-town Drug Store corner of Main and Allen streets, where the choicest articles of the Materea Medica are carefully dispensed, and we have no doubt his beautiful new store and stock will attract his full share of public patronage.

---

MR. WILLIAM KING's old Drug Store in Buffalo makes no changes as we have yet learned. His long and well-established successful business knows no change. It is fully established by long and faithful square dealing, and no alterations need be looked for.

———:o:———

DEATH OF DR. GURDON BUCK, of New York.—Dr. Buck, who has been ill for a long time, died of Uremic Coma, March 6th. He was born May 4th, 1807. He was a well known and successful Surgeon. His achievements were notably in autoplastic Surgery and in the treatment of fractures. We are indebted to him perhaps more than to any other Surgeon for our present simple extension plan of treating fractures of the thigh by means of weight and pulley.

The new edition of the United States Dispensatory will be ready in a few weeks. Part I, has been revised by Dr. George B. Wood. Part II and III, by Dr. H. C. Wood, aided by Prof. Bridges of the Philadelphia College of Pharmacy.—*Record.*

